# Bioactive Peptides–Probiotics Interactions: Implications for Microbial Function and Human Health

**DOI:** 10.3390/foods15060979

**Published:** 2026-03-10

**Authors:** Yue Fan, Qingping Wu, Lanyan Huang, Ying Zhang, Xiuhua Lin, Qihui Gu, Moutong Chen

**Affiliations:** 1 School of Food Science and Engineering, Shaanxi University of Science & Technology, Xi’an 710021, China; fanyue422@sina.com; 2 State Key Laboratory of Applied Microbiology Southern China, Guangdong Provincial Key Laboratory of Microbial Safety and Health, National Health Commission Science and Technology Innovation Platform for Nutrition and Safety of Microbial Food, Key Laboratory of Big Data Technologies for Food Microbiological Safety, State Administration for Market Regulation, Institute of Microbiology, Guangdong Academy of Sciences, Guangzhou 510070, China; wqp203@163.com (Q.W.); lanyan.huang@ecs-scnu.org (L.H.); zhangy9823@163.com (Y.Z.); linxh@gddcm.cn (X.L.); guqh888@163.com (Q.G.); 3 Food and Drug Laboratory, Guangdong Detection Center of Microbiology, Guangzhou 510070, China

**Keywords:** bioactive peptides (BPs), functional foods, gut microbiota, health promotion, lactic acid bacteria (LAB), probiotics

## Abstract

Bioactive peptides (BPs) and probiotics have attracted increasing attention in food and nutrition research for their roles in microbial metabolism and functional food development, with lactic acid bacteria (LAB) representing widely used probiotic microorganisms possessing well-characterized metabolic and peptide transport systems within the gut microbiota. This review summarizes current knowledge on food-derived BPs and their interactions with probiotic LAB, with a particular focus on peptide transport and utilization mechanisms, including oligopeptide permease (Opp) and di-/tripeptide permease (Dpp) systems. Sources and production methods of BPs are reviewed, along with experimental evidence describing peptide-supported microbial growth and metabolic responses. Relevant analytical approaches used for peptide characterization and functional assessment are also discussed. Most available evidence derives from controlled *in vitro* studies and primarily reflects microbial physiological responses rather than direct host-level effects. This review provides a mechanistic perspective on peptide–probiotic interactions in LAB and outlines research directions related to nitrogen utilization and microbial functional performance.

## 1. Introduction

Bioactive peptides (BPs) are short amino acid sequences released from food proteins through enzymatic hydrolysis, microbial fermentation, or gastrointestinal digestion [1,2]. These peptides typically consist of a limited number of amino acid residues and are inactive within the parent protein sequence until released. Depending on their amino acid composition and sequence, BPs have been reported to exhibit a broad spectrum of biological activities, including antimicrobial, antihypertensive (angiotensin-converting enzyme-inhibitory), antioxidant, and immunomodulatory effects, as documented in experimental systems and bioactive peptide databases such as the bioactive polypeptide database (BIOPEP-UWM; https://biochemia.uwm.edu.pl/biopep/peptide_data.phpaccessed on 5 February 2026) [3,4,5,6,7].

In recent years, increasing attention has been paid to the identification of BPs from natural food materials as well as to approaches for their controlled production, driven by growing interest in functional foods and nutrition-based health modulation. Numerous studies have described peptide-mediated biological effects, such as inhibition of *Phytophthora capsici* spore germination by peptides derived from *Brevibacillus laterosporus* [8] or the suppression of pathogenic *Escherichia coli* growth in the presence of soybean-derived peptides that promote *Lactobacillus* proliferation [9]. It should be noted, however, that most reported biological functions of BPs are derived from *in vitro* or animal models, and their physiological relevance in humans remains highly context-dependent.

The human gut microbiota plays a central role in host physiology, influencing metabolic regulation, immune homeostasis, and barrier function. Probiotics are generally defined as live microorganisms that, when administered in adequate amounts, confer health benefit on the host [10,11,12]. Among them, lactic acid bacteria (LAB), including species of *Lactobacillus*, *Lacticaseibacillus*, *Limosilactobacillus*, and *Bifidobacterium*, are widely studied due to their long history of use in fermented foods and their metabolic versatility [13,14].

Alterations in gut microbial composition can contribute either to improved host health or to disease development. Accumulating evidence indicates that microbial metabolites such as short-chain fatty acids (SCFAs) and lipopolysaccharides are involved in metabolic regulation, inflammatory responses, and gut–brain communication [15,16,17,18]. Probiotic interventions have been reported to modulate epithelial barrier integrity, immune signaling, and microbial competition in experimental models [19,20,21]. However, extrapolation of these findings to clinical outcomes should be approached with caution.

Traditionally, prebiotics are defined as selectively utilized substrates, most commonly non-digestible carbohydrates, that confer health benefits through modulation of the gut microbiota [22,23]. These compounds resist host digestion, remain unabsorbed in the upper gastrointestinal tract, and are selectively utilized by beneficial microorganisms in the colon [24,25,26]. However, most dietary peptides are susceptible to enzymatic hydrolysis and absorption in the upper gastrointestinal tract, and only a limited fraction may reach the distal gut. Accordingly, BPs differ from classical prebiotics and generally do not meet the criteria required for classification as carbohydrate-based prebiotic substrates, such as inulin or fructooligosaccharides [27,28,29,30].

It should be noted that probiotic microorganisms can generate peptides through their own metabolic activity; however, these endogenously produced peptides are largely shaped by microbial nutritional demands and are difficult to control. In contrast, food-derived BPs supplied exogenously are present prior to microbial metabolism and may exert additional or distinct modulatory effects under defined experimental conditions.

Instead, accumulating evidence suggests that BPs may act as metabolic modulators and support probiotic growth [9,31,32,33,34,35,36,37,38], adhesion [39], or stress tolerance [40,41] under specific experimental conditions. A variety of peptide structures, dosage, the enzymatic capacity of microorganisms, and the specificity of host microbiome composition govern the effect scale [39,40,41,42,43]. In addition, variability across studies may be the result of differences in preparing and delivering peptides [44].

Peptides–probiotics interactions can be distinguished from carbohydrate-based prebiotics by the peptide transport systems involved, particularly the Opp and Dpp pathways in LAB. To enable appropriate peptide uptake, these systems work by defining length ranges, linking extracellular peptide availability to intracellular metabolism and functional responses [45,46].

The relatively predictable growth-promoting effects observed in controlled experiments can be explained partly by the direct utilization of peptides via the Opp/Dpp transport system that is less dependent on a complex microbial community. This characteristic differentiates these peptides from conventional prebiotics [15,29,47]. Nevertheless, more sufficient validation is required to clarify how such mechanisms contribute to long-term microbiota modulation and host health outcomes in humans.

This review firstly critically examines the BP–probiotics interactions, focusing on peptide utilization through Opp/Dpp transport systems and metabolic pathways in LAB. Second, this review summarizes the reported health benefits and probiotic growth effects driven by nutrient differences, including the sources and production of food-derived BPs. Finally, peptides–probiotics interactions and current limitations or research challenges in related investigations are emphasized.

The focus of this narrative review is the interactions between food-derived peptides and probiotic microorganisms. Relevant literature was identified from Web of Science, PubMed, and Scopus, with most studies published within the last 10–15 years. “Bioactive peptides,” “protein hydrolysates,” “lactic acid bacteria,” “probiotics,” “peptide transport,” “Opp system,” “Dpp system,” “nitrogen utilization,” “stress tolerance,” and “microbial metabolism” were used as keywords in the search. The selected studies have relevance to peptide production, microbial utilization, and probiotic-related outcomes, with conclusions based on available experimental evidence.

The current review provides a mechanistic perspective on BP-involved probiotic growth responses and helps explain variability across studies by integrating peptide sources and preparation methods with microbial transport and utilization. Appropriate caution regarding functional food applications is also maintained.

## 2. Sources and Production of BPs

### 2.1. Natural Sources of BPs and Their Biological Relevance

BPs are widely derived from natural food-related materials, reflecting their close association with human diets and nutritional intake [48,49,50,51,52,53]. Based on their origin, BP sources can be broadly classified into plant-derived [54,55,56,57,58,59,60,61,62], animal-derived [7,63,64,65,66,67,68,69], and fermented or dairy-related materials [32,65,70,71,72,73,74]. These diverse sources give rise to structurally and functionally heterogeneous peptides that underpin their varied biological activities.

Plant-derived materials represent one of the most extensively investigated sources of BPs. Common plant origins include soybean, rice bran, sesame, walnut, wheat, corn gluten meal, and Douchi [75,76,77,78,79,80,81,82]. Peptides generated from these materials have frequently been associated with probiotic-supporting effects, particularly the promotion of *Lacticaseibacillus* and *Bifidobacterium* growth. For example, soybean-derived peptides have been reported to enhance the proliferation of *Lacticaseibacillus rhamnosus* and *Limosilactobacillus reuteri* [83,84], while decapeptides derived from natural rubber serum stimulated *Bifidobacterium bifidum* growth [85]. In addition to growth promotion, certain plant-derived peptides exhibit antimicrobial or antioxidant activities, such as cationic peptides derived from rice bran [86].

Animal-derived materials constitute another major category of BP sources. Aquatic products and poultry by-products have been shown to support the viability and metabolic activity of probiotic strains, including *Lactobacillus plantarum* and *Bifidobacterium* spp. [33,36]. Hydrolysates derived from these substrates often display multifunctional properties, combining growth-promoting, antioxidant, and stress-protective effects.

Dairy-derived and fermented foods are among the most thoroughly studied BP sources. Casein-derived peptides modulate lactic acid production, enzymatic activity, and overall metabolic performance of LAB, while growth-promoting effects on *Bifidobacterium* and *Lactobacillus* have been consistently observed [32]. It is notable that, during cheese fermentation or yogurt digestion, the released peptides retain biological activity after gastrointestinal processing, which supports their usage in food-based applications [74,87].

Beyond source identification, characterization of molecular size, structure, purity, and stability, and the *in vitro* and *in vivo* assessment of biological activity are standard laboratory evaluations of BPs. Diverse biological effects of BPs have been shown in numerous studies, ranging from probiotic growth promotion, antimicrobial activity, and antioxidant capacity to indirect modulation of host physiological responses after dietary intake. The origins of BPs, peptide composition, and physicochemical properties are combined determinants of BP functionality [33,35].

Recent advances in peptide production and analysis have boosted the efficiency and controllability of peptide generation by using enzyme-membrane integration, online size exclusion chromatography-high-performance liquid chromatography (SEC-HPLC) monitoring, and machine learning-assisted enzyme selection. In addition, enzymatic membrane reactors and continuous biotransformation platforms enable the production of peptides with defined properties while sustaining bioactivity [60,66,88,89,90].

Taken together, various natural materials, such as plants, animal by-products, and fermented or dairy-derived foods, are capable of generating BPs. These sources are reservoirs for peptides with biological potential, showing different protein compositions and processing histories.

### 2.2. Technological Approaches to Protein Hydrolysate and Peptide Production

The process of BP production includes appropriate extraction, hydrolysis, and downstream processing. The optimality of methods determines peptide yield, composition, and functional potential, which affects the production efficacy. A prerequisite for subsequent peptide generation is high extraction efficiency with acceptable protein purity, as it directly influences the feasibility of the whole process and product consistency [50]. Accordingly, BP production through various technological approaches aims not only to maximize peptide release but also to preserve structural features associated with bioactivity.

Ultrasound-assisted processing is a physical pretreatment that is widely applied in food systems to enhance protein accessibility before hydrolysis [91,92]. Inducing protein unfolding state and increasing molecular flexibility are two main ways of ultrasound treatment to improve extraction efficiency and facilitate enzymatic cleavage without introducing chemical residues. However, excessive treatment may have a negative effect on protein integrity or may promote undesirable oxidation reactions; therefore, controlled application of such techniques is needed [93].

Microbial fermentation is an alternative route for BP generation through the biotransformation of proteins using complex proteolytic systems [94]. The release of fermentation-mediated peptides has been reported for substrates such as soybean by-products, yielding antimicrobial or antitumor peptides [95]. Nevertheless, systematic process optimization is vital because the diversity and regulation of microbial proteolytic pathways introduce variability in peptide composition [96].

Enzymatic hydrolysis shows satisfactory efficiency, specificity, and industrial compatibility; it remains the most commonly employed strategy for BP production [97]. The enzyme-specific cleavage preferences of different proteases, including Alcalase, pepsin, flavourzyme, and neutral protease, result in generating hydrolysates with distinct peptide profiles, reflecting differences in peptide molecular weight and bioactivity [98,99,100]. Microbes generally prefer smaller peptides for bioactivity expression, and peptide size distribution determines their function, which is often controlled in preparation through ultrafiltration [64,98,101]. To enrich low-molecular-weight peptides that have better antioxidant or probiotic-supporting properties, sequential or combined enzymatic treatments have also been explored to modulate peptide composition [102,103].

Food-derived peptides with different structures may exert distinct biological functions. For example, short cationic and hydrophobic peptides from chia (*Salvia hispanica*) seed proteins obtained by microwave-assisted enzymatic hydrolysis exhibited pronounced antimicrobial activity against foodborne pathogens via membrane disruption, which is fundamentally different from the probiotic growth-promoting effects of nutrient peptides [90].

BP production efficiency and functional relevance are evaluated by comprehensive characterization and quantification. Routine employment of analytical techniques such as reversed-phase high-performance liquid chromatography (RP-HPLC), mass spectrometry (MS), and LC-MS-based peptide profiling to elucidate peptide composition and structure-activity relationships is common [86,94]. Quantitative approaches, such as derivatization-assisted LC-MS methods, provide a sensitive and reproducible assessment of peptide yields [104]. Collectively, these technological and analytical methods form the foundation for linking BP production processes to biological outcomes, facilitating subsequent evaluation of peptides–probiotics interactions, which are summarized in Table 1 and discussed in later sections. Such findings highlight that interactions between dietary small molecules and bacterial surface proteins could be a novel regulation of probiotic tolerance through a peptide-independent pathway [105].

## 3. LAB-Peptides Functional Interactions

### 3.1. LAB Metabolism in Relation to Peptide Transport and Utilization

The nitrogen and amino acid requirements in LAB metabolism rely on efficient peptide acquisition and utilization systems, particularly in growth environments where multiple nitrogen sources, such as beef extract, yeast extract, and peptone, serve as the primary nitrogen supply. In laboratory cultivation, the growth and metabolic activity of LAB are supported by complex media such as MRS (De Man, Rogosa, Sharpe), which provides peptides, free amino acids, and carbohydrates. However, peptide-based nitrogen sources often play a dominant role in sustaining LAB proliferation and functionality both *in vitro* and *in vivo* [45].

**Figure 1:**
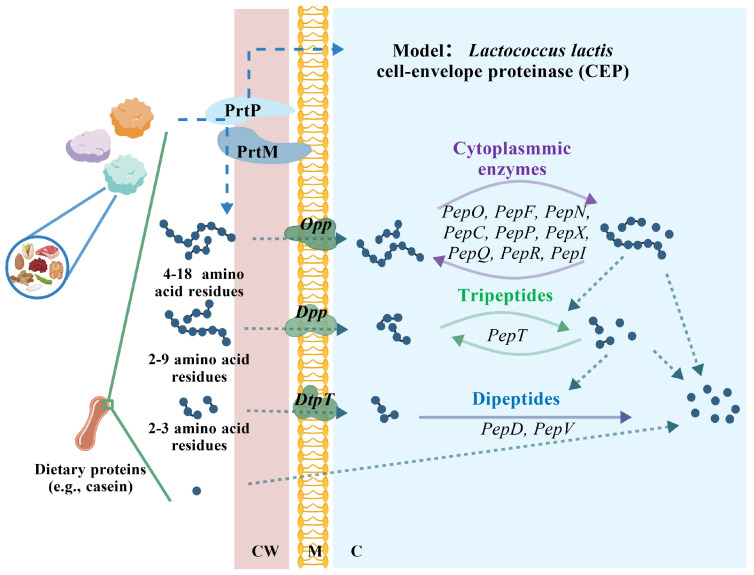
Schematic overview of nitrogen acquisition and peptide utilization in LAB, illustrated using the cell-envelope proteinase (CEP) system of *Lactococcus lactis* as a representative model. Dietary proteins (e.g., casein) are initially hydrolyzed by the cell-envelope proteinase PrtP, assisted by the maturation protein PrtM, generating oligopeptides of different lengths. Peptides containing approximately 4–18 amino acid residues are transported into the cytoplasm via the Opp, whereas dipeptides and tripeptides (2–3 amino acid residues) are taken up mainly through the Dpp and the DtpT. Once inside the cytoplasm, imported peptides are further degraded by a set of intracellular peptidases (including PepO, PepF, PepN, PepC, PepP, PepX, PepR, and PepI) into free amino acids, which are subsequently used for cellular metabolism and protein biosynthesis. CW, cell wall; M, membrane; C, cytoplasm. Solid arrows indicate peptide transport or enzymatic conversion steps, curved arrows represent intracellular metabolic processing, and dashed arrows indicate possible or indirect metabolic pathways.

The LAB proteolytic system, for instance, the *Lactococcus lactis* cell-envelope proteinase (CEP) model as the representative, integrates extracellular protein hydrolysis, peptide transport, and intracellular metabolism (Figure 1) [111]. Dietary proteins, such as casein, are cleaved into oligopeptides of varying lengths by extracellular proteinases. The resulting peptides are transported across the cell membrane via dedicated peptide transport systems.

Peptides containing multiple amino acid residues are taken by the Opp systems, whereas di- and tripeptides are internalized through Dpp and Dtp transporters, respectively. This well-allocated transport strategy enables LAB to capture nitrogen sources from complex protein substrates efficiently.

After internalization, peptides are further hydrolyzed by cytoplasmic peptidases to release free amino acids required for cellular activities such as growth, stress resistance, and metabolic homeostasis. Experimental evidence demonstrates impaired LAB growth due to the disruption of peptide transport systems, particularly Opp, highlighting the preferential utilization of oligopeptides over free amino acids or di-/tripeptides in the tested LAB species/strains. Conversely, robust proliferation and metabolism of strains with intact proteolytic and transport systems confer competitive advantages in nutrient-limited environments.

Activation of peptide-based nitrogen metabolism also plays a role in LAB stress tolerance and ecological fitness. Improved adaptation to acidic environments and stronger biofilm formation by enhanced proteolytic activity indirectly strengthen LAB-mediated inhibition of pathogenic microorganisms. Moreover, the efficient uptake and utilization of peptides are closely linked to industrial traits, including fermentation performance, flavor development, and product quality. Above all, transport and utilization systems of peptides in LAB constitute a central metabolic hub through which BPs are converted into functional advantages in both nutrient supplements and host-associated environments.

### 3.2. Peptide-Derived Modulation of Probiotic Activity

The nitrogen content in LAB and its probiotic activity are influenced by peptide availability, which, accordingly, affects LAB growth, metabolism, stress tolerance, and competitiveness. The more efficient utilization of peptides, especially oligopeptides, by LAB peptide transport systems, rather than intact proteins or free amino acids, leads to increased biomass and metabolic activity [2,112,113,114].

The promotion of LAB growth by peptide-derived nitrogen sources under laboratory and simulated gastrointestinal conditions is shown in experimental studies. Organic acid production in *Limosilactobacillus reuteri* is enhanced by the presence of digested soybean proteins and peptides, thereby strengthening its antibacterial activity [84]. Food-derived oligopeptides give LAB a growth advantage over *Escherichia coli* in co-culture systems, which has been linked to preferential peptide uptake and higher metabolic activity [9,84]. These results indicate that peptides function as effective growth substrates.

Stress responses in LAB and their environmental adaptability are peptide-affected. Enhanced peptide utilization increases biofilm formation and the production of extracellular polymeric substances, which improve tolerance to acidic conditions, bile salts, and heat [115,116]. Furthermore, maintaining intracellular amino acid balance under changing nutrient conditions is achieved by regulating protease and peptidase expression [45].

**Figure 2:**
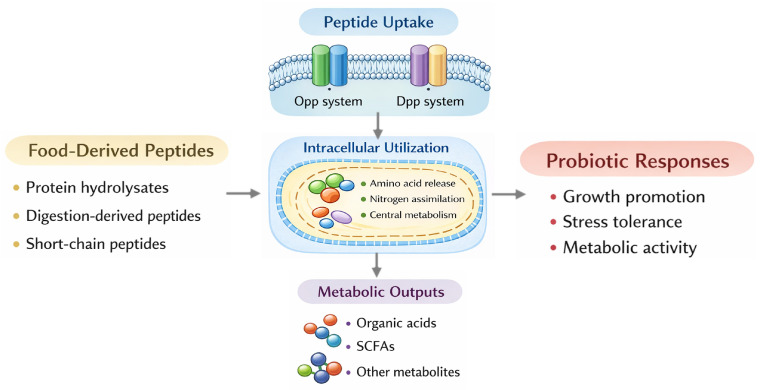
Schematic overview of peptide-mediated metabolic responses in LAB. Food-derived peptides, including protein hydrolysates, digestion-derived peptides, and short-chain peptides, enter LAB cells through Opp and Dpp systems, enabling peptide uptake. Following cellular entry, peptides undergo intracellular utilization involving amino acid release, nitrogen assimilation, and central metabolic pathways. These processes lead to the production of metabolites such as organic acids and SCFAs, contributing to probiotic physiological responses such as growth promotion, stress tolerance, and enhanced metabolic activity.

The performance of LAB in food and in the host is further influenced by peptide metabolism. Low-molecular-weight peptides derived from mushroom basal bulbs exhibited *in vitro* activation of alcohol dehydrogenase and aldehyde dehydrogenase, indicating a host enzyme-targeted bioactivity, which is distinct from peptides–probiotics interactions [117]. Improving peptide utilization is usually associated with the overall optimization of the microbiome, including better fermentation efficiency, altered metabolism for desired flavor production, and increased resistance to pathogen-related stress. Also, peptide-driven changes in metabolic by-products can affect the growth of neighboring microorganisms, contributing to shifts in microbial composition and changes at the community level [27]. The mechanisms of peptide uptake and intracellular utilization in LAB are summarized in Figure 2.

### 3.3. Structural Determinants and Peptide Composition Preferences of BP Functions

The structure of BPs in LAB is strongly related to their functional outcomes, which determine peptide transport efficiency, metabolic utilization, and downstream biological effects. Peptide molecular weight, charge distribution, amino acid composition, and sequence motifs are key parameters that shape the interactions among peptides, bacterial transport systems, and intracellular metabolic machinery [115,118,119].

The preference for peptide utilization is mainly determined by molecular size. Low-molecular-weight peptides show more efficient transport than intact proteins. However, excessive hydrolysis into free amino acids may reduce competitive uptake advantages. Experimental evidence indicates that, under certain growth conditions, LAB preferentially utilize oligopeptides over di- or tripeptides, which is consistent with the dominant role of oligopeptide permease systems in nitrogen acquisition [120]. To generate functionally relevant peptide pools, improvements and modifications to controlled hydrolysis methods should be based on this size-dependent preference.

Charge properties and amino acid composition have an influence on peptide-associated functions as well. Hydrophobic residues are commonly linked to antioxidant activity, while cationic peptides often display antimicrobial effects by disrupting bacterial membranes [118]. Sequence-specific features, such as peptides containing defined motifs, contribute to immunomodulatory and metabolic responses through selectively interacting with cellular targets or regulatory pathways [115,119]. Consequently, variations in peptide sequence can result in distinct metabolic responses and functional outputs. A richness in specific amino acids with hydrophobic or branched-chain residues has been recognized as an important characteristic in developing functional bioactive peptides [91]. For example, papain-hydrolyzed silver carp peptides promoted the growth of *Bifidobacterium animalis* by providing sulfhydryl-containing amino acids and peptides [36], while *Lacticaseibacillus rhamnosus* and *bifidobacteria* exhibited utilization preferences depending on peptide structure or specific motifs in the sequence [83,87]. In some cases, defined peptide sequences may influence microbial or host metabolic pathways through enzyme modulation; however, such effects remain context-dependent and require more evidence [121].

Importantly, the variety of peptide utilization preferences across bacterial species and strains reflects differences in transport capacity and metabolic specialization. For example, faster growth of *Streptococcus mutans* and *Streptococcus sanguis* on hydrophilic peptides than on hydrophobic substrates—one possible explanation could be the less efficient utilization of hydrophobic peptides due to limitations in membrane transport or intracellular processing [79]. Similarly, strain-specific adaptation to peptide-based substrates also occurred in *Lacticaseibacillus rhamnosus* and *Bifidobacterium* species, which display selective growth responses depending on peptide structural features [36,83,91].

Beyond growth effects, peptide structural attributes may influence host-associated metabolism through indirect microbial mechanisms, although current evidence remains limited. Overall, BP utilization and function depend on peptide size, charge, sequence composition, and strain-specific transport and metabolic capacity, involving coordinated processes of proteolysis, transport, and intracellular utilization rather than simple nitrogen supply. Based on this framework, the following section summarizes experimental evidence on the effects of BPs and peptide-derived hydrolysates on probiotic growth and functionality, organized by experimental model and strength of evidence.

## 4. Recent Advances in the Effects of BPs on Probiotic Growth

Recent studies have reported that BPs can support probiotic growth primarily by serving as accessible sources of nitrogen and energy for microbial metabolism [122]. Probiotic species, particularly *Lactobacillus* and *Bifidobacterium*, possess limited amino acid biosynthesis pathways and therefore exhibit strong dependence on exogenous oligopeptides for proliferation [122]. Compared with intact proteins or free amino acids, short peptides have been shown to represent more efficient substrates for probiotic growth [38]. Representative studies examining the effects of food-derived bioactive peptides on probiotic growth and functional responses are summarized in Table 1. Studies are categorized according to the degree of peptide characterization to distinguish purified peptides from defined fractions and complex hydrolysates.

At the mechanistic level, the presence of well-developed peptide transport and proteolytic systems, including the Opp and Dpp pathways, enables rapid uptake and utilization of di- to oligopeptides [123]. This metabolic capacity has been proposed to underlie the observed increases in biomass accumulation, substrate utilization efficiency, and production of fermentation metabolites such as lactic acid and SCFAs under *in vitro* fermentation conditions [122].

Experimental evidence further indicates that peptide characteristics, including molecular weight and amino acid composition, influence probiotic growth responses. Ding et al. reported that a low-molecular-weight walnut oligopeptide enriched in Glu, Asp, Arg, and Leu significantly enhanced the proliferation of *Lactobacillus plantarum* Z7 in a dose-dependent manner [124]. In addition to increased viable cell counts, supplementation with this peptide preparation was associated with elevated extracellular polymeric substance production and biofilm formation, accompanied by reduced bacterial mortality under thermal, thermotolerance, acidic, or bile stress conditions [110,124,125,126].

Beyond single-strain models, multiple *in vivo* and *in vitro* gastrointestinal studies have reported increased abundance of *Lactobacillus* and *Bifidobacterium* following supplementation with peptide preparations derived from dairy proteins, soybean, sesame meal, fish, and meat by-products [36,75,84,122]. For example, peptides derived from Parmigiano-Reggiano cheese were associated with enhanced bifidobacterial and lactobacilli proliferation, whereas sesame meal peptides promoted *Lactobacillus* abundance while suppressing *Escherichia coli* in poultry gut ecosystems [87]. Similar growth-supporting effects have been reported for peptide hydrolysates derived from natural rubber serum and keratin, which enhanced the proliferation of multiple probiotic species [77,85,127,128,129].

Collectively, these findings suggest that BPs can influence probiotic growth through diverse mechanisms, including the provision of amino acid substrates, stimulation of bacterial metabolic activity, and the enhancement of stress resilience. However, most reported effects are derived from controlled experimental systems, and BPs appear to function primarily as growth-supporting substrates rather than as classical prebiotics. Consequently, their role in probiotic modulation should be interpreted mainly in the context of microbial-level responses rather than as definitive evidence for direct host health outcomes [130].

## 5. LAB-BPs Interactions and Their Implications for Human Health

### 5.1. Effects of BPs on Probiotic Growth and Microbial Interactions

Accumulating evidence indicates that supplementation with BPs can influence the growth and metabolic activity of LAB, primarily by serving as accessible nitrogen sources and modulating bacterial physiological states [9,32,84,131]. The enhanced growth performance of *Lactobacillus* and *Bifidobacterium* strains has been observed in several studies using protein hydrolysates or peptide-enriched media, usually along with increased production of metabolites and altered gene expression profiles [84,131].

Peptide molecular weight is considered a key factor in determining bacterial utilization efficiency. Short peptides, particularly those with molecular weights below 1 kDa, have been reported to support LAB growth in a concentration-dependent manner [32,116]. For example, promoted proliferation under controlled cultivation conditions was exhibited by the enhancement of nitrogen utilization efficiency in *Limosilactobacillus reuteri* due to soybean-derived peptides [84]. These observations show consistency with the established preference of LAB for oligopeptides over intact proteins or free amino acids [32].

In addition to probiotic growth promotion, BPs may influence microbial interactions within mixed communities. Using *in vitro* or simulated gastrointestinal systems, suppression of opportunistic pathogens such as *Escherichia coli* has been observed through the accumulation of organic acids associated with peptide utilization [9,84]. Moreover, certain peptide preparations have been shown to promote biofilm formation and adhesion capacity of probiotics, which may improve bacterial persistence under environmental stress [116,132]. It should be noted that such effects primarily reflect advantages of microbial competition, which is indirect inhibition of pathogens, rather than direct antimicrobial activity [133,134,135].

Of particular interest is the influence of peptides on probiotic growth, which is one of the critical roles of bioactive compounds. Different classes of bioactives act through distinct mechanisms. Compounds such as sesamin, anthocyanins, and dietary fibers also have the ability to modulate probiotic abundance by affecting adhesion-related proteins or microbial enzymatic activities [136,137,138]. While these studies provide useful background insights, extra caution is required in the application of using non-peptide effects to assist peptide-specific mechanisms.

Overall, according to current evidence, BPs act as modulators of probiotic growth and microbial interactions; this dynamic is the result of altered nutrition and metabolism. Most findings are derived from *in vitro* or animal-based studies, and their relevance to complex human gut ecosystems depends on dosage, peptide composition, and microbiota [43,93,139].

### 5.2. Modulation of Gut Microbiota and Functional Delivery of BPs and Probiotics

Sufficient quantities of BPs in bioactive form must reach the gut for them to exert measurable biological effects within the gastrointestinal tract. The stability, solubility, and delivery efficiency of peptides and probiotics are improved via techniques such as encapsulation and complexation to adapt to the gastrointestinal environment [86,140,141]. For instance, probiotics have been co-encapsulated with plant-derived extracts to enhance bacterial survival and facilitate intestinal delivery in experimental models [140].

Peptide-involved modulation of gut microbial composition has been observed in both animal studies and limited human interventions. The administration of protein hydrolysates or peptide-enriched diets has shown shifts toward increased abundance of beneficial bacterial taxa, along with a reduction in opportunistic pathogens [139,142]. These microbial changes are often accompanied by alterations in metabolite profiles; for instance, increased production of SCFAs is associated with gut barrier maintenance and immune modulation [32,143].

Nevertheless, caution is required during the interpretation of these outcomes. Specific modulation of microbial functions can lead to microbiota shifts; however, in some cases, the changes in the microbiome may also reflect enhanced probiotic viability or metabolic competitiveness. Furthermore, peptide utilization is not exclusive to beneficial microbes, and the possibility that certain pathogenic bacteria may also access peptide-derived nutrients cannot be excluded [144]. Building a clear balance between probiotic support and unwanted microbial utilization is essential for correct peptides–probiotics usage.

Variability in peptide composition, lack of standardized dosing instructions, and limited clinical validation are possible factors that constrain the application of the BP-probiotic system. Although the investigation of BPs-mediated improvement of probiotic resilience under acidic or bile stress conditions is provided, evidence for consistent host-level health benefits remains insufficient [143,145,146,147].

In summary, gut microbiota modulation by BPs is mainly achieved through supporting probiotic survival and metabolism. Gut microbiota dysbiosis is characterized by reduced microbial diversity and altered community structure, often leading to impaired intestinal barrier function and excessive immune activation [15,16]. Disruption of epithelial integrity facilitates the translocation of microbial components such as lipopolysaccharides, which can trigger innate immune responses and promote chronic inflammation [148]. In contrast, probiotics and their metabolites, particularly SCFAs, help restore immune homeostasis by enhancing epithelial barrier function, promoting regulatory T cell (Treg) differentiation, and suppressing pro-inflammatory cytokine production [149]. Through modulation of Treg/Th17 balance and inhibition of NF-κB signaling pathways, probiotic-derived metabolites contribute to the prevention or attenuation of inflammatory diseases, including inflammatory bowel disease and metabolic disorders [150]. However, current data largely support indirect and context-dependent effects, emphasizing the need for well-controlled human studies to clarify their functional significance within complex host-microbiome systems [149,151,152,153].

## 6. Current Challenges and Research Limitations

Although extensive research has demonstrated the applicability of BPs in various fields, several critical issues remain unresolved. It is still uncertain whether BPs with a specific amino acid sequence can be produced selectively by existing enzymatic or microbial fermentation methods, rather than by chemical synthesis. Although chemical synthesis enables highly precise sequence control and rational modification of peptides [154,155,156], major challenges remain in terms of large-scale production cost, particularly when translating optimized sequences into practical applications. Furthermore, the potential differences between naturally derived and chemically synthesized BPs have not been thoroughly clarified, particularly regarding their structure-function relationships and biological activities. During fermentation, the accumulation of organic acids may also influence the stability or bioactivity of BPs. Another unexplored area is whether pathogenic bacteria could competitively utilize BPs rather than probiotics, thereby influencing probiotic growth or host-microbe interactions [2,157]. The mechanisms underlying BP-pathogen interactions remain largely unknown [158]. Finally, it is important to consider whether the amount of probiotics and their metabolites exceeds a physiological tolerance threshold, which would lead to unpredictable effects on host homeostasis. Integrated studies combining advanced peptide synthesis, omics-based metabolic profiling, and microbial ecology can address these challenges by providing a comprehensive understanding and control of BPs–microbe–host interactions [159,160,161]. These are stepwise and evidence-driven methods that clarify when and how BPs-probiotic interactions may be applied in real and general treatments, rather than supporting broad functional claims.

## 7. Conclusions and Future Perspectives

Key considerations in exploring peptides–probiotics combinations are peptide molecular size, availability as nitrogen sources, and compatibility with microbial transport systems, as suggested by *in vitro* data.

BPs derived from natural food sources have emerged as functional dietary components. Their capability of interacting with probiotic microorganisms through microbial metabolic and transport-related mechanisms is the primary functional mechanism. In LAB, the close link among BPs–probiotics interactions, the peptide uptake systems, nitrogen utilization efficiency, and microbial stress adaptation is supported by increasing experimental evidence.

However, several issues limit the current understanding of BPs–probiotics interactions. First, most reported effects are derived from controlled *in vitro* systems or animal models, which raises uncertainty about extending these findings to complex human gut ecosystems. Moreover, direct comparison across studies is difficult because of variability in peptide structure, production methods, microbiota composition, and host physiological conditions.

Addressing these limitations mechanistically and providing standardized approaches should be the focus of future research. Elucidating structure-function relationships of peptides related to microbial utilization, establishing protocols of production and characterization that are reproducible, and defining dose–response relationships under physiological conditions should be considered priorities. Comparison of naturally derived and chemically synthesized peptides may clarify the sequence-specific or context-dependent properties of BP bioactivities in detail.

It is important to interpret BP-mediated modulation of probiotic activity at the microbial level by *in vivo* or clinical investigation. Well-designed animal studies and human intervention trials are necessary to determine the feasibility of applying BP-related microbial responses to consistent and significant host-level outcomes, with ensured safety and tolerability.

In summary, the ability of BPs to modulate probiotic behavior and microbial metabolism confers on them a promising role as diet-derived compounds. Applying BPs in functional foods or health-related products will require more mechanistic validation, cautious interpretation of research data, and a clear clarification of effects at the microbial level and overall host health conditions. Continued interdisciplinary efforts integrating peptide chemistry, microbial ecology, and systems-level analyses will be essential to advance this field in a scientifically robust and translationally responsible manner.

## Figures and Tables

**Table 1:** Effects of food protein-derived peptides on probiotic growth and metabolism *in vitro*.

Peptide Source	Preparation/Production	Peptide Fraction/Features	Characterization Level	Target Probiotic Strain(s)	Evidence Types	Main Observed Effects	Mechanistic Clue	References
Soybean	Simulated GI digestion (pepsin + pancreatin)	Short-chain and hydrophilic peptides enriched	Level III—Complex hydrolysate	*Lacticaseibacillus rhamnosus* Lra05	*In vitro*	Growth promotion and metabolic activation	Preferential utilization of short-chain peptides (mechanism not fully elucidated)	[83,106]
Soybean	Enzymatic hydrolysis/simulated digestion of soybean protein	RP-HPLC fractionated peptides; specific short peptides identified (e.g., LISPL, IQLP, IAANPA, FASPA, IATSPA, IIP)	Level I—Purified peptides (defined sequences)	*Limosilactobacillus reuteri* LR08	*In vitro*	Enhanced probiotic growth and organic acid secretion; synergistic growth promotion with FOS	Nitrogen-source peptides enhance probiotic metabolism and act synergistically with carbon-source prebiotics	[107]
Soybean	Digested soybean proteins (dpro) and digested soybean peptides (dpep)	Not specified	Level III—Complex hydrolysate	*Limosilactobacillus reuteri* LR08	*In vitro* (co-culture and inhibition zone assays)	Promoted growth and metabolism of *L. reuteri* under competition with *E. coli*; enhanced competitiveness	Increased organic acid secretion and improved nitrogen utilization capacity	[84]
Walnut protein	Alkaline protease hydrolysis of defatted walnut dregs	PPNKW (PW5, 100 μg/mL)	Level I—Purified peptide (defined sequence)	*Lacticaseibacillus rhamnosus* LGG	*In vitro*	Reversed growth inhibition caused by PS500 microplastics	Formation of PW5-PS500 complex via hydrogen bonding and van der Waals interactions, reducing MP inhibitory effects	[43,108]
Parmigiano Reggiano cheese	Simulated gastrointestinal digestion (oral, gastric, duodenal)	Digestion-derived peptides (2–24 amino acids); 71 new peptides identified	Level II—Defined peptide fraction	*Bifidobacterium* (27 strains); *Lactobacillus* (30 strains)	*In vitro* (pure cultures and human colonic microbiota batch cultures)	Promoted growth of *bifidobacteria* and most *lactobacilli*; higher growth on PR digests than on control peptone	Strain- and species-specific peptide utilization preferences; differential peptide consumption patterns between bifidobacteria and lactobacilli	[87]
Poultry by-products (bone and meat)	Enzymatic hydrolysis (e.g., 78T)	Hydrolysates rich in free amino acids; no specific peptide sequences reported	Level III—Complex hydrolysate	*Lactobacillus* spp. (ten strains)	*In vitro* (growth kinetics in supplemented media)	Supported maximum growth rate and biomass yield to MRS; in some cases, superior to tryptone or peptone	High free amino acid content supplies auxotrophic requirements	[35]
Poultry processing leftovers (meat and feathers)	Enzymatic hydrolysis (FPAP, FFP)	Characterized by total/soluble nitrogen, molecular weight distribution, and free amino acids; no specific sequences reported	Level III—Complex hydrolysate	*Lactobacillus* spp.; *Bifidobacterium* spp.	*In vitro* (growth media supplementation; microscopy)	Promoted growth and maintained viability	Nutritional supplementation supporting biomass formation	[33]
Caseinomacropeptide (κ-casein-derived)	Pepsin treatment (simulated gastric digestion)	κ-casein fragment f (106–124); low-pH-active peptide;	Level I—Purified peptide (defined fragment)	*Lactobacillus rhamnosus*	*In vitro*	Increased acid resistance (pH 3.5); no growth promotion	Protective effect under acidic conditions;	[41]
Chickpea	Enzymatic digestion of chickpea protein	Albumin-derived peptide fraction; high antioxidant activity	Level II—Defined peptide fraction	*Bifidobacterium* spp.; LAB (e.g., *Pediococcus, Weissella*); *Veillonella*	*In vitro* (fecal batch fermentation)	Promoted *bifidobacteria* growth; enhanced SCFA production; reduced ammonia and indole formation	Antioxidant activity associated with modulation of colonic fermentation	[109]
*Cordyceps militaris* mycelium	Extraction of albumin and glutenin followed by enzymatic hydrolysis	Glutenin-derived peptides, 5–10 kDa; identified peptide MR-10 (MAVNLVPFPR)	Level I—Purified peptide (MR-10 identified)	*Lacticaseibacillus paracasei* R21	*In vitro* (heat stress model, microscopy, multi-omics)	Enhanced thermoprotection and survival (65 °C); increased viable cell counts; improved membrane integrity	MR-10 is involved in peptide transport via ABC transporters; promoted biofilm formation, and fatty acid synthesis	[110]

Peptides are classified based on the level of characterization: Level I (purified peptides with defined sequences), Level II (defined peptide fractions), and Level III (complex hydrolysates or digests).

## Data Availability

No new data were created or analyzed in this study. Data sharing is not applicable to this article.

## Abbreviations

The following abbreviations are used in this manuscript:

BPs	Bioactive peptides
LAB	Lactic acid bacteria
*E. coli*	*Escherichia coli*
MW	Molecular weight
SCFAs	Short-chain fatty acids
Opp	Oligopeptide permease
Dpp	Di-/tripeptide permease
RP-HPLC	Reversed-phase high-performance liquid chromatography
SEC-HPLC	Size exclusion chromatography-high-performance liquid chromatography
